# Predictors of Liver Steatosis Progression Among People Living With HIV in Low‐ and Middle‐Income Countries: A Multicentre Longitudinal Study

**DOI:** 10.1002/jia2.70184

**Published:** 2026-09-29

**Authors:** Marie K. Plaisy, Thierry Tiendrebeogo, Carlotta Mondoka, Mark H. Kuniholm, Rodrigo de Carvalho Moreira, Aggrey Semeere, Rohidas T. Borse, Albert Minga, Gilles Wandeler, Gad Murenzi, Brenda E. Crabtree‐Ramírez, Niharika Samala, Jeremy Ross, Ephrem Mensah, Ardele Mandiriri, Sandra W. Cardoso, Jean Paul Mivumbi, Lameck Diero, Haridas Prasad, Paola Alarcón Murra, Belinda V. Chihota, Suzanne Goodrich, Thida Chanyachukul, Ellen Brazier, Stephany N. Duda, Hugo Perazzo, Antoine Jaquet, the International epidemiology Databases to Evaluate AIDS (IeDEA)

**Affiliations:** ^1^ Research Institute for Sustainable Development (IRD) EMR 271, Bordeaux Population Health Centre Bordeaux France; ^2^ Department of Infectious Diseases Bern University Hospital Bern Switzerland; ^3^ Institute of Social and Preventive Medicine University of Bern Bern Switzerland; ^4^ Department of Epidemiology and Biostatistics University at Albany State University of New York Albany New York USA; ^5^ Evandro Chagas National Institute of Infectious Diseases ‐Oswaldo Cruz Foundation (INI/FIOCRUZ) Rio de Janeiro Brazil; ^6^ Infectious Diseases Institute Makerere University Kampala Uganda; ^7^ BJ Government Medical College and Sassoon General Hospital Pune India; ^8^ Blood Bank Medical Centre, the HIV Care Clinic of the National Blood Transfusion Center Abidjan Côte d'Ivoire; ^9^ Research for Development (RD Rwanda) and Rwanda Military Referral and Teaching Hospital Kigali Rwanda; ^10^ Departamento De Infectología, Instituto Nacional De Ciencias Médicas y Nutrición Salvador Zubirán Mexico City Mexico; ^11^ Division of Gastroenterology and Hepatology Department of Medicine Indiana University Indianapolis Indiana USA; ^12^ TREAT Asia, amfAR ‐ The Foundation for AIDS Research Bangkok Thailand; ^13^ NGO Espoir‐Vie Togo Lomé Togo; ^14^ Newlands Clinic Harare Zimbabwe; ^15^ Department of Medicine School of Medicine College of Health Sciences Moi University Eldoret Kenya; ^16^ Institute for Implementation Science in Population Health City University of New York New York New York USA; ^17^ Department of Biomedical Informatics Vanderbilt University Medical Center Nashville Tennessee USA

**Keywords:** HIV infection, liver steatosis, low‐and middle‐income countries, MASLD, risk factors, steatosis progression

## Abstract

**Introduction:**

Liver steatosis is increasingly recognized as a major comorbidity in people living with HIV (PLWH), with growing evidence suggesting an increased risk of progression to advanced liver disease. We assessed predictors of liver steatosis progression over 36 months in PLWH from low‐ and middle‐income countries.

**Methods:**

Between 2020 and 2023, we enrolled PLWH aged ≥40 years who had been on antiretroviral therapy (ART) for ≥6 months at nine HIV clinics in the IeDEA Sentinel Research Network (SRN) across the Asia‐Pacific, the Americas and African IeDEA regions, and followed them prospectively for 36 months. Participants with available and reliable controlled attenuation parameter (CAP) measurements at both enrolment and the 36‐month visits were included in the analysis. Steatosis progression was defined as incident steatosis (CAP ≥248 dB/m) or a transition to a higher steatosis grade (moderate ≥268 dB/m; severe ≥280 dB/m) among those with a lower steatosis grade at enrolment. Logistic regression was used to assess predictors of progression.

**Results:**

We included 923 participants (63% female; median age 50 years, interquartile range 45–55) in this analysis. At enrolment, 51% were overweight or obese, 10% had type 2 diabetes mellitus (T2DM), 94% had HIV RNA <1000 copies/mL and 72% were on integrase strand inhibitor (INSTI)‐based ART. During the 36‐month follow‐up, 190 (20%) participants showed steatosis progression, including 147 (74%) with incident steatosis and 49 (26%) with worsening of steatosis grades. The progression rate was significantly higher in Asia‐Pacific (33% [95% CI, 24−44]) and the Americas (36% [95% CI, 26−46]) compared to the African regions (17% [95% CI, 14−20]). Independent predictors of steatosis progression were overweight/obesity (aOR: 2.97, 95% CI: 2.03−4.40), T2DM (aOR: 2.41, 95% CI: 1.45−3.96) and dyslipidaemia (aOR: 1.46, 95% CI: 1.01−2.12). In females, INSTI use for ≥2 years at enrolment was additionally associated (aOR 3.19, 95% CI: 1.35−8.01).

**Conclusions:**

Metabolic disorders—including T2DM, overweight/obesity and dyslipidaemia—were associated with steatosis progression in PLWH in our cohort. These findings underscore the need for targeted monitoring and prevention strategies to address this significant public health issue.

## 1 Introduction

Liver steatosis is a leading cause of liver disease‐related morbidity and mortality in people living with HIV (PLWH) worldwide, with an estimated prevalence of approximately 38% [1]. A meta‐analysis, predominantly conducted in high‐income countries, reported a prevalence of 34% for liver steatosis and 12% for moderate liver fibrosis in PLWH without viral hepatitis infection, based on non‐invasive imaging techniques [2]. In low‐ and middle‐income countries (LMICs), we previously reported an overall steatosis prevalence of 28%, with higher rates observed in Asia‐Pacific and Latin America, and lower rates in Africa [3].

Liver steatosis is often considered a benign condition, but long‐term follow‐up shows that about 9% of individuals with simple steatosis may progress to advanced fibrosis and end‐stage liver disease [4]. Although the majority of individuals with liver steatosis will never experience serious liver‐related complications, its high prevalence poses a significant public health concern, contributing to substantial economic burden and an increasing demand for liver transplantation [5].

Liver steatosis encompasses a spectrum of histopathological features, ranging from simple steatosis to steatohepatitis, with or without fibrosis, which may further progress to advanced stages, including cirrhosis and end‐stage liver disease [6]. In PLWH without viral hepatitis co‐infection, liver steatosis is of particular clinical concern due to its faster progression and greater severity [7]. This accelerated progression is likely driven by several factors, including direct viral effects on lipogenesis, hepatotoxic effects of antiretroviral therapy (ART), frequent metabolic disorders and chronic inflammation [8, 9]. Given that liver steatosis is a chronic process, longitudinal studies are needed to evaluate the disease dynamic and factors influencing its progression among PLWH, particularly those without viral hepatitis co‐infections.

Metabolic disorders such as obesity, dyslipidaemia and type 2 diabetes mellitus (T2DM) are well‐established risk factors for liver steatosis worldwide [10, 11, 12, 13, 14, 15]. Recently, the presence of steatosis with at least one cardiometabolic risk factor, without hazardous alcohol consumption, defined metabolic‐associated steatotic liver disease (MASLD) [16]. In addition, exposure to certain nucleoside reverse‐transcriptase inhibitors (NRTIs) such as stavudine (D4T) and didanosine (DDI) has been associated with liver steatosis through mitochondrial toxicity mechanisms [11, 14, 17, 18, 19]. Similarly, certain non‐nucleoside reverse transcriptase inhibitors (NNRTIs), especially efavirenz, have been shown in vitro to induce lipid accumulation in hepatocytes [20, 21], with cumulative exposure to NNRTIs associated with steatosis progression in PLWH with hepatitis C virus (HCV) co‐infection [22]. Protease inhibitors (PIs) have been shown to contribute to liver steatosis through insulin resistance and hyperlipidaemia [23, 24]. More recently, integrase strand transfer inhibitors (INSTIs) have gained attention for their association with weight gain and metabolic abnormalities, which may also contribute to the occurrence of liver steatosis [25, 26, 27].

Despite this accumulating evidence, most of these data are from cross‐sectional studies, which limits understanding of the dynamics of liver steatosis. Longitudinal studies assessing the natural history and predictors of liver steatosis are scarce, particularly in low‐resource settings. Existing cohort studies of liver steatosis progression have largely focused on PLWH with HCV co‐infection or those in high‐income countries [22, 28, 29, 30].

In this study, we aimed to assess the progression of liver steatosis─encompassing both new cases of steatosis and progression to a higher grade─and to identify its predictors over 36 months of follow‐up among PLWH, without viral hepatitis co‐infection in LMICs.

## 2 Methods

### 2.1 Study Design and Population

This study utilized data from the Sentinel Research Network (SRN), a prospective cohort within the International epidemiology Databases to Evaluate AIDS (IeDEA) consortium. The SRN includes PLWH aged ≥40 years and on antiretroviral therapy (ART) ≥6 months, recruited at HIV clinics in 12 countries across six global IeDEA regions: Asia‐Pacific (*n* = 2: India, Vietnam), Caribbean, Central and South America (CCASAnet) (*n* = 2: Brazil, Mexico), Central Africa (*n* = 1: Rwanda), East Africa (*n* = 3: Kenya, Tanzania, Uganda), Southern Africa (*n* = 2: Zambia, Zimbabwe) and West Africa (*n* = 2: Côte d'Ivoire, Togo) enrolled from October 2020 to October 2023. SRN participants were recruited during their routine HIV care visit using two sampling approaches: systematic random sampling for sites with a large number of eligible PLWH, or consecutive enrolment for those with a small number of eligible PLWH [31]. Participants undergo baseline (SRN enrolment), 6‐, 12‐, 24‐ and 36‐month study visits (±1 month), during which standardized questionnaires, laboratory tests and review of medical records are conducted to assess various chronic conditions.

In this study, we included participants from nine HIV clinics across the six global IeDEA regions: Asia‐Pacific (India), CCASAnet (Brazil and Mexico), Central Africa (Rwanda), East Africa (Kenya), Southern Africa (Zambia and Zimbabwe) and West Africa (Côte d'Ivoire and Togo) that performed vibration‐controlled transient elastography (VCTE) at the SRN baseline visit and the month 36 visit.

### 2.2 Data Collection

Standardized questionnaires were used to collect sociodemographic information such as age and sex at birth. The Alcohol Use Disorders Identification Test (AUDIT) assessed hazardous alcohol consumption (score ≥8 in males and ≥7 in females) [32]. Measured weight and height were used to calculate body mass index (BMI) = weight (kg)/[height (m)]^2^. BMI ≥25 kg/m^2^ (23 Asia) and ≥30 kg/m^2^ (25 Asia) were defined as overweight and obesity, respectively [33]. Central obesity was defined with a waist circumference ≥94 cm for males and ≥80 cm for females at sites in Africa and ≥90 cm for males and ≥80 cm for females at sites in Asia and CCASAnet [34]. Blood pressure (BP) was measured three consecutive times using an automated cuff. Hypertension was defined based on a mean diastolic BP ≥90 mmHg or mean systolic BP ≥140 mmHg or any prescribed anti‐hypertensive medication [35]. Fasting glycaemia, HbA1c and a lipid panel including total cholesterol, Low‐Density Lipoprotein (LDL), High‐Density Lipoprotein (HDL) and triglycerides were measured in plasma at each annual SRN visit. T2DM was defined as glucose ≥ 7.0 mmol/L or HbA1c ≥ 6.5% or any history of treatment for T2DM [36]. Dyslipidaemia was defined as total cholesterol ≥6.20 mmol/L, triglycerides >2.25 mmol/L, LDL >4.13 mmol/L or HDL <1.03 mmol/L for males or <1.29 mmol/L for females [37]. Co‐infections with hepatitis B virus (HBV) and HCV were assessed using rapid diagnostic tests on participants’ blood samples (Determine, SD Bioline, or Abon for HBs antigen and Oraquick or SD Bioline for anti‐HCV antibodies). Aspartate aminotransferase (AST), alanine aminotransferase (ALT) and platelet count were also systematically measured. HIV viral load and CD4 cell counts were measured during SRN visits. If these measures were not available on the day of the SRN visit, the nearest available HIV viral load and CD4 values were obtained from medical records. Information related to past and current ART regimens was also extracted from participants’ medical records. ART regimens at enrolment were classified as ART anchor (INSTI, NNRTI, PI) and ART backbone (abacavir [ABC], tenofovir alafenamide [TAF], tenofovir disoproxil fumarate [TDF]).

### 2.3 Liver Evaluation and Outcome

Liver fibrosis and steatosis were assessed by liver stiffness measurement (LSM) and controlled attenuation parameter (CAP) by VCTE (FibroScan, EchoSens, Paris, France), respectively. VCTE examinations were performed by trained operators following a standardized protocol, with participants fasting for at least 3 h. Two FibroScan models were used (502 or Expert 630). An XL probe was available at five of the nine study sites, with a switch from an M probe to an XL probe based on the device's automatic selection tool. VCTE results were deemed reliable when the following criteria were fulfilled: (i) ≥10 successful measurements; (ii) interquartile range (IQR) <30% of median CAP for steatosis and LSM for fibrosis; and (iii) success rate >60% with the FibroScan 502 model [38]. Only participants with reliable CAP measurements at both baseline and 36 months were included in this analysis.

Significant liver fibrosis was defined as LSM ≥7.1 kPa [39]. Steatosis was classified according to CAP thresholds: no steatosis (S0) <248 dB/m, mild steatosis (S1): 248–267 dB/m, moderate steatosis (S2): 268–279 dB/m and severe steatosis (S3): ≥280 dB/m [40]. Steatosis progression was defined as the development of any grade of steatosis in participants without steatosis at SRN enrolment, or a transition to a higher grade among those with a lower grade at enrolment.

### 2.4 Covariates

Covariates were selected a priori using directed acyclic graphs (DAGs) developed for each main predictor of interest. Adjustment sets varied across models and included metabolic and ART‐related factors, while age, sex and region were included in all models as fixed confounders. The corresponding DAGs are provided in the Supplementary Figure S1.

### 2.5 Statistical Analysis

We described characteristics of participants at baseline according to steatosis status (no progression vs. progression) using median and IQR for continuous variables and counts and percentages for categorical variables.

Factors at enrolment associated with liver steatosis progression over the 36‐month follow‐up were assessed using logistic regression models. Univariable analyses were first performed, followed by multivariable models adjusted for plausibly true confounders (age, sex, region) and other confounders identified through a DAG‐based approach.

For missing values (<5%) in TAF, PI and NNRTI variables, assumed to be missing at random (MAR), simple imputation using the mode was applied [41]. Variables not considered MAR, such as dyslipidaemia, were not imputed to avoid bias. Given evidence of greater weight gain in females compared with males after switching to INSTI [42, 43], we performed an exploratory sex‐stratified analysis to assess the potential impact of prior INSTI exposure on steatosis progression.

Results were reported as adjusted odds ratio (aOR) with 95% confidence interval (CI). Analyses were performed using R software Version 4.4.1.

### 2.6 Ethical Considerations

All participants provided written informed consent before being included. Institutional review boards at each site approved the study protocol (INI‐Fiocruz, Brazil: 28609820.9.0000.5262; CMSDS, Côte d'Ivoire:195‐21; BJ Medical College, India: 00241024; AMPATH Eldoret, Kenya: IREC/2020/41; INCMNSZ, Mexico: 3708; Kicukiro Health Center, Rwanda: 885/RNEC/2022; EVT Clinic, Togo: 01/2022/CBRS; CIDRZ, Zambia: 00001131 of IORG0000774; Newlands Clinic, Zimbabwe: MRCZ/A/2475.

## 3 Results

A total of 1862 PLWH, aged ≥ 40 years on ART for at least 6 months, were enrolled and completed the 36‐month follow‐up. Overall, 939 individuals did not meet the inclusion criteria and were excluded, mainly due to absent or unreliable CAP measurements at enrolment and/or the 36‐month visits, hazardous alcohol consumption, chronic HBV or HCV co‐infection, or missing data on HIV viral load, CD4 count and ART cumulative duration (Figure 1).

**FIGURE 1:**
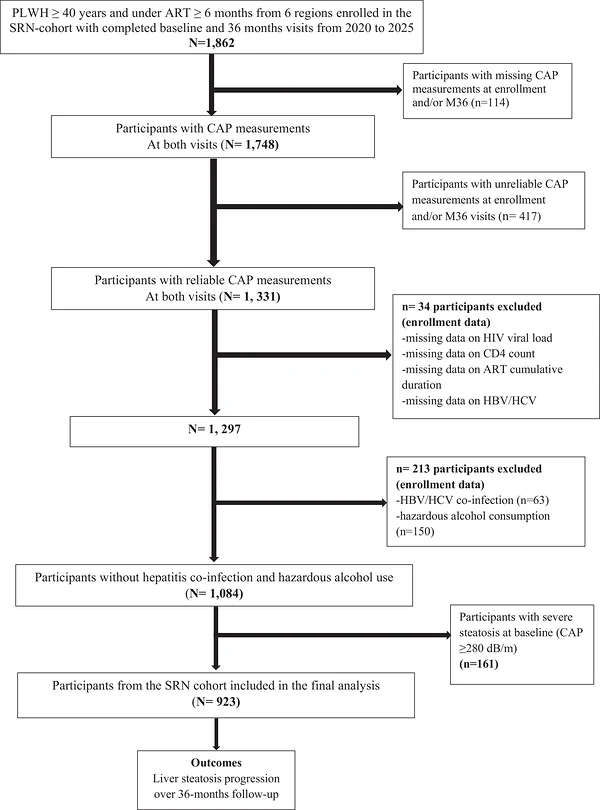
Flow chart of the selection of the study population.

The final study population included 923 participants from six regions: Asia‐Pacific (*n* = 87), Central Africa (*n* = 294), CCASAnet (*n* = 95), East Africa (*n* = 104), Southern Africa (*n* = 127) and West Africa (*n* = 216). The median age was 50 years (IQR: 45–55), and 63% were female sex. At baseline, 51% were overweight/obese, of whom 75% were female, and 51% had dyslipidaemia. Participants had been on ART for a median duration of 12 years (IQR: 7–15), with a median CD4 cell count of 540 cells/mm^3^ (IQR: 379–712); 6% had an unsuppressed HIV viral load (>1000 copies/mL). At baseline, 72% of participants were receiving an INSTI‐based anchor regimen, with 50% for less than 2 years, and 79% were on a TDF‐containing backbone regimen (Table 1).

**TABLE 1:** Baseline characteristics of the study population according to steatosis progression (*N* = 923).

Characteristic	All (*N* = 923)	No steatosis progression *N* = 733 (80%)	Steatosis progression *N* = 190 (20%)
**Age (IQR) (years)**	50 (45−55)	50 (45−56)	50 (45−54)
**Sex at birth**			
Female	581 (63%)	467 (64%)	114 (60%)
Male	342 (37%)	266 (36%)	76 (40%)
**Body mass index (kg/m^2^)**			
<25^a^	450 (49%)	391 (53%)	59 (31%)
≥25	473 (51%)	342 (47%)	131 (69%)
**Type 2 diabetes** ^b^			
No	832 (90%)	678 (92%)	154 (81%)
Yes	91 (10%)	55 (8%)	36 (19%)
**Hypertension** ^c^			
No	680 (74%)	541 (74%)	139 (73%)
Yes	243 (26%)	192 (26%)	51 (27%)
**HDL cholesterol**			
Normal	459 (50%)	377 (51%)	82 (43%)
Hypo HDL	387 (42%)	294 (40%)	93 (49%)
Missing	77 (8%)	62 (8%)	15 (8%)
**Total cholesterol**			
Normal	834 (91%)	664 (91%)	170 (89%)
High	66 (7%)	49 (7%)	17 (9%)
Missing data	23 (2%)	20 (3%)	3 (1.58%)
**LDL cholesterol**			
Normal	784 (85%)	626 (85%)	158 (83%)
High	55 (6%)	40 (5%)	15 (8%)
Missing data	84 (9%)	67 (9%)	17 (9%)
**Triglycerides**			
Normal	810 (88%)	646 (88%)	164 (86%)
High	85 (9%)	63 (8%)	22 (11%)
Missing data	28 (3%)	24 (3%)	4 (2.11%)
**Dyslipidaemia** ^d^			
No	381 (41%)	315 (43%)	66 (35%)
Yes	473 (51%)	359 (49%)	114 (60%)
Missing data	69 (7%)	59 (8%)	10 (5%)
**Abdominal obesity** ^e^			
No	358 (39%)	312 (42%)	46 (24%)
Yes	565 (61%)	421 (57%)	144 (76%)
**Current tobacco use**			
Never smoked	642 (69%)	520 (71%)	122 (64%)
No	182 (20%)	143 (19%)	39 (20%)
Yes	99 (11%)	70 (9%)	29 (15%)
**ALT**	21 (16−27)	20 (15−27)	23 (17−32)
Unknown	1	1	0
**AST**	25 (21−31)	25 (21−31)	24 (20−31)
Unknown	1	1	0
**GGT**	39 (28−65)	36 (28−56)	63 (27−73)
Unknown	836	671	165
**CAP median**	213 (188−239)	208 (184−236)	232 (210−249)
**Steatosis class**			
Absence^f^	754 (82%)	613 (83%)	141 (74%)
≥S1 (mild steatosis)	113 (12%)	79 (11%)	34 (18%)
≥S2 (moderate steatosis)	56 (6%)	41 (5%)	15 (8%)
**Significant fibrosis** ^g^			
No^h^	790 (93%)	639 (94%)	151 (90%)
Yes	55 (7%)	39 (6%)	16 (10%)
**Duration of ART**			
Median (Q1−Q3)	12 (7−15)	11 (7−15)	12 (8−15)
**ART anchor**			
INSTI	668 (72%)	534 (73%)	134 (71%)
NNRTI	101 (11%)	73 (10%)	28 (15%)
PI	67 (7%)	55 (6%)	12 (6%)
Other	87 (9)	71 (10)	16 (8)
**ART backbone**			
ABC	48 (5%)	45 (6%)	3 (1%)
TAF	13 (1%)	11 (1%)	2 (1%)
TDF	731 (79%)	574 (78%)	157 (82%)
Other	131 (14%)	103 (14%)	28 (15%)
**Past D4T use**			
No	663 (72%)	533 (73%)	130 (68%)
Yes	260 (28%)	200 (27%)	60 (31%)
**Past DDI use**			
No	892 (96%)	710 (97%)	182 (96%)
Yes	31 (3%)	23 (3%)	8 (4%)
**Nadir CD4^+^**	184 (100−288)	180 (96−277)	207 (106−308)
Missing	4	3	1
**HIV RNA viral load**			
Unsuppressed^i^	54 (6%)	46 (6%)	8 (4%)
Suppressed	869 (94%)	687 (94%)	182 (96%)
**CD4^+^ T lymphocyte count**	540 (379−712)	519 (363−687)	607 (455−826)
**Region**			
Asia Pacific	87 (9%)	58 (8%)	29 (15%)
Central Africa	294 (32%)	239 (32%)	55 (29%)
CCASAnet	95 (10%)	61 (8%)	34 (18%)
East Africa	104 (11%)	88 (12%)	16 (8%)
Southern Africa	127 (14%)	104 (14%)	23 (12%)
West Africa	216 (23%)	183 (25%)	33 (17%

Abbreviations: ABC, abacavir; ART, antiretroviral therapy; CCASAnet, Caribbean, Central and South America (Brazil, Mexico); Central Africa (Rwanda); East Africa (Kenya); NNRTIs, non‐nucleoside reverse transcriptase inhibitor; PI, protease inhibitor; Southern Africa (Zambia, Zimbabwe); TAF, tenofovir alafenamide; TDF, tenofovoir disoproxil fumarate. Asia‐Pacific (India); West Africa (Côte d'Ivoire, Togo).

^a^ <23 kg/m^2^ for Asian populations.

^b^ Assessed with glucose ≥ 7.0 mmol/L or HbA1c ≥ 6.5% or any history of treatment for type 2 diabetes.

^c^ Defined as mean systolic BP ≥140 mmHg and/or diastolic BP ≥ 90 mmHg or any prescribed anti‐hypertensive medication.

^d^ Assessed with total cholesterol≥6.20 mmol/L, triglycerides>2.25 mmol/L, LDL>4.13 mmol/L or HDL<1.03 mmol/L for males or <1.29 mmol/L for females.

^e^ Assessed with waist circumference ≥94 cm for males and ≥80 cm for females at sites in Africa and ≥90 cm for males and ≥80 cm for females at sites in Asia and CCASAnet.

^f^ Controlled attenuation parameter (CAP) <248 dB/m.

^g^ Assessed in participants with IQR LSM/median <30%.

^h^ Liver stiffness measurement (LSM) <7.1 kPa.

^i^ Viral load >1000 copies/mL.

Overall, 190 (20% [95% CI, 18−23]) participants experienced liver steatosis progression over the 36‐month follow‐up (Figure 2), of whom 141 (74%) had no steatosis at baseline (Table 1). The highest proportion of progression were observed in the Asia‐Pacific (33% [95% CI, 24−44]) and CCASAnet regions (36% [95% CI, 26−46]), whereas lower proportions were observed in East Africa (15% [95% CI, 9.3−24]) and West Africa regions (15% [95% CI, 11−21]) (Figure 2). Of the 169 participants with liver steatosis at enrolment, 96 (57%) showed regression, with the highest proportion observed in East Africa (73%) and lowest in CCASAnet (35%) (data not shown).

**FIGURE 2:**
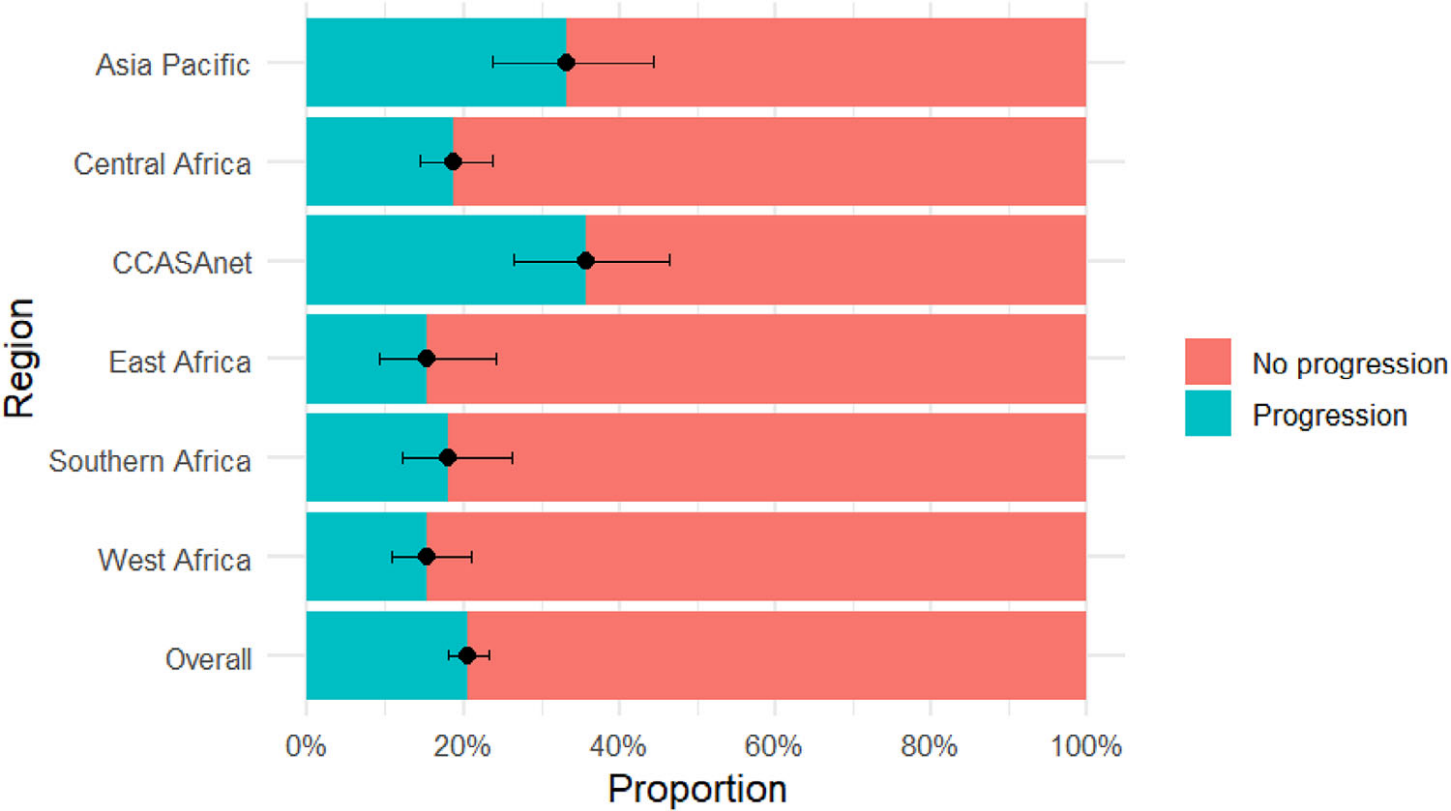
Proportion of steatosis progression over 36 months, overall and by region.

### 3.1 Baseline Characteristics of PLWH by Liver Steatosis Progression

PLWH with liver steatosis progression were more likely to be overweight or obese (69% vs. 46%), have central obesity (76% vs. 57%), T2DM (19% vs. 8%) and dyslipidaemia (60% vs. 49%) compared to those without liver steatosis progression. They also had a higher median CD4^+^ T lymphocyte count (607 vs. 519 cells/mm^3^) and higher ALT levels (median 23 [IQR: 17−32] U/L vs. 20 [IQR: 15−27] U/L) (Table 1).

### 3.2 Predictors of Liver Steatosis Progression

After adjustment, liver steatosis progression was significantly associated with baseline overweight or obesity (aOR 2.97, 95% CI: 2.03−4.40), T2DM (aOR 2.41, 95% CI 1.45−3.96) and dyslipidaemia (aOR 1.46, 95% CI 1.01−2.12). Overall, INSTI exposure was not associated with steatosis progression (Table 2). However, in exploratory sex‐stratified analyses, female participants with ≥2 years of INSTI exposure at enrolment had higher odds of steatosis progression than those unexposed to INSTI (aOR 3.19, 95% CI: 1.35−8.01) (Table S1).

**TABLE 2:** Crude and adjusted logistic regression models of predictors of liver steatosis progression over 36 months period (*N* = 923).

	Crude steatosis progression, OR (95% CI)	Adjusted steatosis progression, OR (95% CI)
**Baseline characteristics**		
Age^a^ (years)	1.00 0.72−1.37	0.88 0.70−1.11
Sex^b^ (male vs. female)	1.17 0.84−1.62	1.01 0.71−1.42
BMI^c^ [<25^d^ vs. ≥25 kg/m^2^]	2.54 1.82−3.59	2.97 2.03−4.40
Hypertension^e^ [Yes vs. No]	1.03 0.71−1.48	0.89 0.58−1.35
Type 2 diabetes^f^ [Yes vs. No]	2.88 1.82−4.53	2.41 1.45−3.96
Dyslipidaemia^g^ [Yes vs. No]	1.52 1.08−2.13	1.46 1.01−2.12
Dyslipidaemia^g^ [Missing vs. No]	0.81 0.37−1.60	1.34 0.53−3.17
**Cumulative exposure to INSTI (years)** ^h^		
Not‐exposed	Ref	Ref
<1	0.93 0.60−1.45	1.51 0.82−2.83
1−2	0.75 0.47−1.17	1.34 0.71−2.56
≥2	1.02 0.65−1.59	1.65 0.87−3.17
**Cumulative ART duration (years)** ^i^		
<10	Ref	Ref
10−15	1.02 0.69−1.49	1.11 0.74−1.65
≥15	1.05 0.70−1.57	1.18 0.76−1.84

Abbreviation: OR, odds ratio.

^a^ Adjusted by sex, region and baseline steatosis.

^b^ Adjusted by age, region and baseline steatosis.

^c^ Adjusted by age, sex, region, baseline steatosis, ART duration and current and past ART exposure (INSTI, PI, NNRTI, TAF, DDI, D4T).

^d^ <23 kg/m^2^ for Asian populations.

^e^ Defined as mean systolic BP ≥140 mmHg and/or diastolic BP ≥ 90 mmHg or any prescribed anti‐hypertensive medication, and model was adjusted by age, sex, region, BMI, baseline steatosis, ART duration and current and past ART exposure (INSTI, PI, NNRTI, TAF, DDI, D4T).

^f^ Assessed with glucose ≥ 7.0 mmol/L or HbA1c ≥ 6.5% or any history of treatment for type 2 diabetes, model was adjusted by age, sex, region, BMI, baseline steatosis, ART duration and current and past ART exposure (INSTI, PI, NNRTI, TAF, DDI/D4T).

^g^ Assessed with total cholesterol≥6.20 mmol/L, triglycerides>2.25 mmol/L, LDL>4.13 mmol/L or HDL<1.03 mmol/L for males or <1.29 mmol/L for females), model was adjusted by age, sex, region, BMI, baseline steatosis, ART duration and current and past ART exposure (INSTI, PI, NNRTI, TAF, DDI, D4T).

^h^ Adjusted by age, sex, region, baseline steatosis, ART duration and current and past ART exposure (NNRTI, PI, TAF, DDI, D4T).

^i^ Adjusted by age, sex, region and baseline steatosis.

## 4 Discussion

In this multiregional analysis conducted in nine HIV clinics across nine LMICs, we found that liver steatosis progressed among one in five PLWH without hepatitis co‐infection over 36 months of follow‐up, with significant regional differences. We identified several factors associated with the progression of liver steatosis, including overweight/obesity, T2DM and dyslipidaemia. Moreover, in exploratory analyses, ≥2 years of INSTI exposure in female participants was associated with this progression.

Longitudinal studies assessing liver steatosis progression among PLWH, particularly in LMICs, remain scarce. Most previous studies have focused on PLWH with HCV co‐infection in high‐income settings. For instance, in a U.S. cohort of 222 PLWH with HCV co‐infection assessed with paired liver biopsies, 26% experienced steatosis progression, slightly higher than our findings [30]. Similarly, a Spanish study of 146 PLWH with HCV, also using paired liver biopsies, reported higher progression rates of 40% over a median follow‐up of 3 (IQR 2–5) years, comparable with the proportion observed in the Asia‐Pacific and CCASAnet regions [22]. A Canadian cohort of 232 PLWH, including individuals with and without HCV, assessed by VCTE with CAP, reported an overall progression rate of 42%, with higher rates among those without hepatitis co‐infection (approximately 60%) [7]. However, to our knowledge, no prospective study has specifically assessed liver steatosis progression among PLWH without hepatitis co‐infection in LMICs.

In contrast to previous studies, our study focused exclusively on PLWH without hepatitis co‐infection, and found marked differences in steatosis progression between regions. Several factors may account for these disparities, including differences in genetic (particularly PNPLA3 I148M polymorphism) and metabolic susceptibility across populations [44, 45]. Additionally, dietary differences and the higher prevalence of metabolic disorders, including high BMI and T2DM, in Asia and Latin America [46], may contribute to these regional disparities.

More than half of participants with baseline steatosis showed regression over 36 months, likely reflecting true metabolic improvement, supported by reduction in BMI and waist circumference, as well as measurement variability. These findings highlight the dynamic nature of steatosis and may influence interpretation of longitudinal progression and serial monitoring.

In our study, we observed that metabolic disorders were significantly associated with liver steatosis progression, in agreement with previous studies. Two longitudinal studies among PLWH with HCV co‐infection identified obesity as the strongest predictor of steatosis progression [7, 30], and a prospective study in PLWH without viral hepatitis infections similarly confirmed this association [47]. Obesity is a well‐established risk factor for both the development of steatosis and its progression to more advanced liver outcomes, including steatohepatitis and cirrhosis [48]. Pathophysiological mechanisms include visceral adipose tissue expansion, adipocyte hypertrophy and macrophage infiltration, leading to insulin resistance, chronic inflammation and dysregulated adipokine secretion (increased leptin, decreased adiponectin), resulting in hepatic accumulation of lipotoxic lipids in obese adults [49, 50]. These interrelated processes contribute to liver steatosis and its progression to more severe liver outcomes [51].

Our findings also showed that T2DM was a predictor of steatosis progression. Steatosis is independently associated with T2DM and is a strong predictor of liver‐related morbidity and mortality [52, 53, 54]. Additionally, a strong correlation has been observed between steatosis severity and metabolic syndrome features, including glucose intolerance [55, 56, 57]. T2DM reflects advanced metabolic dysfunction with β‐cell failure and chronic hyperglycaemia [58].

In our study, dyslipidaemia was independently associated with steatosis progression. This finding is consistent with previous evidence suggesting an association between lipid abnormalities and liver disease progression among PLWH [7, 59]. Both T2DM and dyslipidaemia contribute to liver fat accumulation and disease progression through mechanisms involving altered lipid metabolism, increased inflammatory signalling and oxidative stress [60]. These findings emphasize the need for systematic screening and management of metabolic disorders, including lifestyle interventions such as weight loss, to prevent the development and progression of liver steatosis in PLWH.

Beyond metabolic factors, ART may also contribute to liver steatosis development and progression. Previous studies have highlighted associations between certain ART regimens, particularly PI, TAF and INSTI, and liver steatosis, likely mediated by ART‐related weight gain [26, 47, 61, 62]. For instance, the ADVANCE trial reported substantial weight gain with dolutegravir, especially in combination with TAF/emtricitabine [61]. Similarly, an observational cohort of U.S. women with HIV reported increases in weight and BMI over the first 2 years of follow‐up, along with a higher incidence of steatosis within the first year after INSTI initiation [28]. A German cohort study also found that INSTI and TAF were associated with a higher risk of steatosis development and faster progression of steatosis grade over time [47].

In our study, we did not find any significant association between ART regimens and steatosis progression in the overall analysis. However, in an exploratory sex‐stratified analysis, female participants with ≥2 years of INSTI exposure at enrolment showed higher odds of steatosis progression, a pattern not observed in males. These findings should be interpreted cautiously given their exploratory nature, and may reflect heterogeneity in susceptibility to the metabolic effects of INSTI in females, potentially mediated by greater weight gain and increased visceral adiposity [27, 63].

The lack of association between baseline INSTI exposure and steatosis progression in the overall study population may partly reflect the timing of the first CAP assessment relative to ART initiation or switch. Most participants were already on INSTI‐based ART at their initial CAP assessment, limiting the ability to capture early changes in weight gain and steatosis severity that may occur shortly after INSTI initiation or switch. In addition, steatosis progression may have been influenced by INSTI initiation or withdrawal between baseline and M36, not captured by baseline ART exposure. In contrast, the U.S. and German cohorts followed participants from ART initiation or switch, allowing a more comprehensive assessment of these drug‐related effects.

Taken together, our findings reinforce the role of metabolic disorders as primary drivers of liver steatosis progression among PLWH in LMCs, supporting a holistic approach to HIV care, integrating systematic screening, preventive strategies, and comprehensive management of metabolic disorders and liver steatosis.

Among the limitations of our analysis, the study population consisted of PLWH aged ≥40 on ART, which may have contributed to higher outcome rates and limited generalizability of our findings to the broader population of PLWH in the participating countries. Lifestyle factors including diet and genetic polymorphisms known to influence steatosis progression were not assessed. In addition, CAP measurements were not aligned with ART initiation or switch, which may have limited our ability to detect early metabolic effects of INSTI‐based regimen on steatosis progression. Consequently, the potential impact of ART exposure, including INSTI‐based regimens, may have been underestimated. Defining INSTI exposure based on duration at enrolment may introduce survivorship bias and confounding by indication, limiting causal interpretation of the observed sex‐stratified associations. Given evidence that liver steatosis may appear within the first year following INSTI initiation or switch, future studies using an emulated trial design or randomized clinical trials with synchronized CAP assessments and treatment changes would be more appropriate to further investigate these associations. Moreover, the exclusion of participants with missing or unreliable CAP measurements at both visits may have introduced selection bias and limited the generalizability of our findings. Missing CAP measurements were more common among participants with overweight or obesity requiring XL probes, likely reflecting limited XL probe availability, whereas unreliable CAP measurements were primarily driven by elevated IQRs, reflecting measurement variability. Finally, steatosis progression was assessed using a fixed‐interval definition between baseline and 36 months of follow‐up, limiting evaluation of longitudinal trajectories of liver fat accumulation. Overall, these limitations underscore the challenges related to the implementation and longitudinal use of CAP to diagnose liver steatosis in LMIC cohorts.

Despite these limitations, our study provides unique insights to assess predictors of liver steatosis progression over 36 months of follow‐up using data from a large multicentre cohort study across six global regions and nine LMICs. Other strengths include standardized data collection, VCTE examinations performed by trained operators and the use of CAP cutoffs for steatosis classification based on a meta‐analysis correlating CAP values with histologic steatosis grades in the general population [40]. An additional strength might be the ability to exclude participants with hazardous alcohol consumption and viral hepatitis infection based on validated instruments and a blood draw, and data collection by REDCap.

## 5 Conclusions

In conclusion, metabolic disorders, including overweight/obesity, T2DM and dyslipidaemia, were found to be the main predictors of liver steatosis progression among PLWH in LMICs. While INSTI‐based regimens may contribute to this progression through weight gain in female participants, no association was reported in our overall study population. Additional observational and experimental studies are required, aligned with treatment initiation or switching, and with longer follow‐up. Enhancing the prevention and management of metabolic disorders should be a key priority in long‐term HIV care to reduce the risk of liver steatosis and its progression to severe complications.

## Author Contributions

*Conceptualization*: MKP and AJ. *Data management*: MKP. *Statistical analysis*: MKP. *Writing – original draft*: MKP. *Writing – revising and editing*: MKP and AJ. *Critical review for important intellectual content and approval of the final manuscript*: TT, CM, MHK, RCM, AS, RTB, AM, GW, GM, BEC‐R, NS, JR, EM, AM, SWC, JPM, LD, HP, PAM, BVC, SG, TC, EB, SND and HP.

## Funding

The International epidemiology Databases to Evaluate AIDS (IeDEA) is supported by the U.S. National Institutes of Health's (NIH), National Institute of Allergy and Infectious Diseases, the *Eunice Kennedy Shriver* National Institute of Child Health and Human Development, the National Cancer Institute, the National Institute of Mental Health, the National Institute on Drug Abuse, the National Heart, Lung, and Blood Institute, the National Institute on Alcohol Abuse and Alcoholism, the National Institute of Diabetes and Digestive and Kidney Diseases, and the Fogarty International Center: Asia‐Pacific, U01AI069907; CCASAnet, U01AI069923; Central Africa, U01AI096299; East Africa, U01AI069911; Southern Africa, U01AI069924; West Africa, U01AI069919. Informatics resources are supported by the Harmonist project, R24AI124872.

## Conflicts of Interest

The authors declare no conflicts of interest.

## Disclaimer

This work is solely the responsibility of the authors and does not necessarily represent the official views of any of the institutions mentioned above. This work is subject to the NIH Public Access Policy. Through acceptance of this federal funding, NIH has been given the right to make this manuscript publicly available in PubMed Central upon the Official Date of Publication, as defined by NIH.

## Supporting information




**Supporting File**: jia270184‐sup‐0001‐SuppMat.docx

## Data Availability

Data may be made available in line with the IeDEA standard operating procedures for data‐sharing.
