# Supplementary material for: Predictors of Liver Steatosis Progression Among People Living With HIV in Low‐ and Middle‐Income Countries: A Multicentre Longitudinal Study
Source: J Int AIDS Soc. 2026 Sep 29;29(10):e70184. doi: 10.1002/jia2.70184 (PMC13623011; doi:10.1002/jia2.70184)
Supplement: Supplementary file 1 [file JIA2-29-e70184-s001.docx]

**Supplementary Table:** Crude and adjusted logistic regression models of predictors of liver steatosis progression over 36-months period stratified by sex (*N* = 923)

|  | **Crude steatosis progression,**  **OR* (95% CI)** | **Adjusted steatosis progression,**  **OR* (95% CI)** |
| --- | --- | --- |
| ***Body Mass Index (BMI)*** |  |  |
| ***Female sex*** |  |  |
| BMI^a^ [<25^b^ vs ≥25 kg/m^2^] | 3.36 2.07 – 5.65 | 3.79 2.24 – 6.67 |
| ***Male sex*** |  |  |
| BMI^a^ [<25^b^ vs ≥25 kg/m^2^] | 2.32 1.38 – 3.92 | 2.40 1.30 – 4.47 |
| ***Cumulative exposure to INSTI (years)****^c^* |  |  |
| ***Female sex*** |  |  |
| Not-exposed | Ref | Ref |
| <1 | 1.22 0.70 – 2.13 | 1.96 0.88 – 4.57 |
| 1-2 | 1.03 0.57 – 1.86 | 1.79 0.76 – 4.42 |
| ≥2 | 1.56 0.87 – 2.81 | 3.19 1.35 – 8.01 |
| ***Male sex*** |  |  |
| Not-exposed |  |  |
| <1 | 0.63 0.29 – 1.33 | 1.17 0.41 – 3.38 |
| 1-2 | 0.46 0.22 – 0.90 | 0.79 0.29 – 2.22 |
| ≥2 | 0.54 0.27 – 1.07 | 0.63 0.22 – 1.83 |

^a^Adjusted by age, region, baseline steatosis, ART duration and current and past ART exposure (INSTI, PI, NNRTI, TAF, DDI, D4T).

^b^<23 kg/m^2^ for Asian populations.

^c^Adjusted by age, region, baseline steatosis, ART duration and current and past ART exposure (NNRTI, PI, TAF, DDI, D4T).

**Abbreviation:** *, Odds Ratio

a)

b)

c)

e)

f)

d)

**Supplementary Figure:** Directed Acyclic Graphs (DAGs) illustrating the assumed causal structure for the multivariable models.

a) Model 1: Exposure = BMI ≥25 kg/m^2^; b) Model 2: Exposure =HTA; c) Model 3: Exposure=Type 2 diabetes; d) Model 4: Exposure=Dyslipidemia;

e) Model 5: Exposure=INSTI exposure, f) Model 6: Exposure=Cumulative ART duration.

*Outcome for all models = Steatosis progression.

Note: Age, sex, and region were considered as fixed confounders and were not represented in the DAGs.
